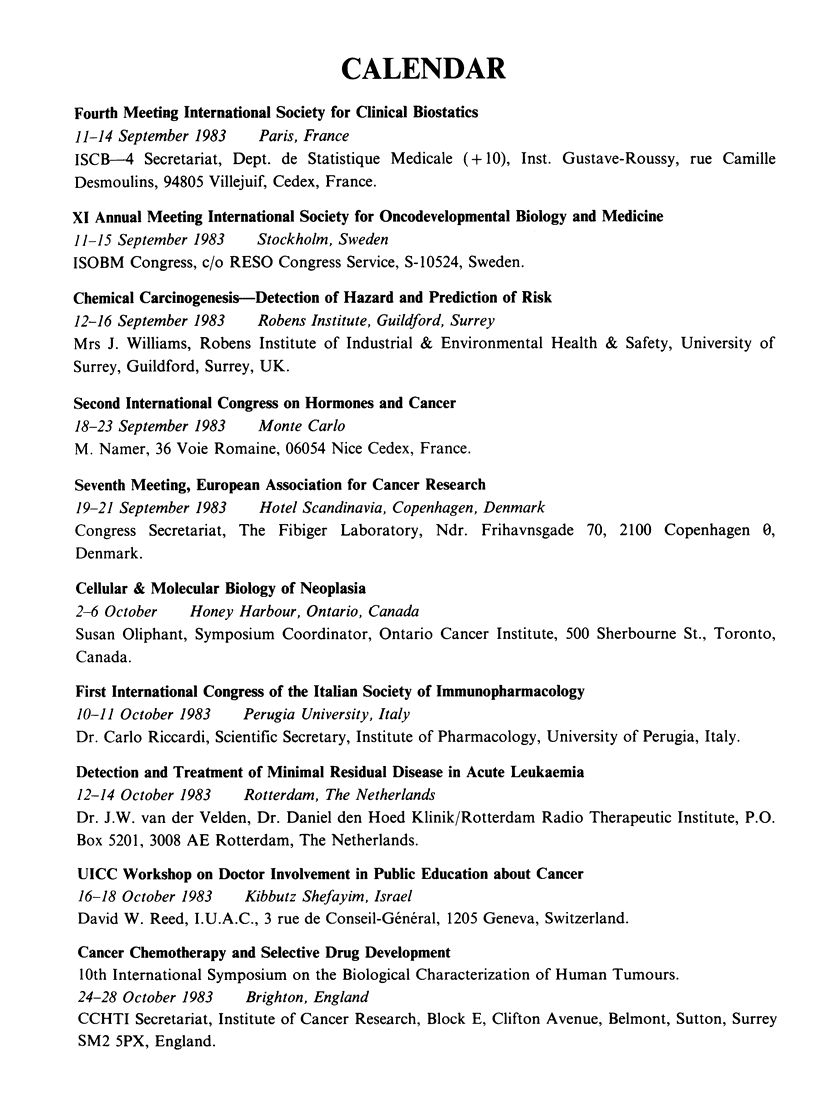# Calendar

**Published:** 1983-08

**Authors:** 


### CALENDAR

**Fourth Meeting International Society for Clinical Biostatics**
*11–14 September 1983* *Paris, France*
ISCB—4 Secretariat, Dept. de Statistique Medicale (+10), Inst. Gustave-Roussy, rue Camille Desmoulins, 94805 Villejuif, Cedex, France.

**XI Annual Meeting International Society for Oncodevelopmental Biology and Medicine**
*11–15 September 1983* *Stockholm, Sweden*
ISOBM Congress, c/o RESO Congress Service, S-10524, Sweden.

**Chemical Carcinogenesis—Detection of Hazard and Prediction of Risk**
*12–16 September 1983* *Robens Institute, Guildford, Surrey*
Mrs J. Williams, Robens Institute of Industrial & Environmental Health & Safety, University of Surrey, Guildford, Surrey, UK.

**Second International Congress on Hormones and Cancer**
*18–23 September 1983* *Monte Carlo*
M. Namer, 36 Voie Romaine, 06054 Nice Cedex, France.

**Seventh Meeting, European Association for Cancer Research**
*19–21 September 1983* *Hotel Scandinavia, Copenhagen, Denmark*
Congress Secretariat, The Fibiger Laboratory, Ndr. Frihavnsgade 70, 2100 Copenhagen Ø, Denmark.

**Cellular & Molecular Biology of Neoplasia**
*2–6 October* *Honey Harbour, Ontario, Canada*
Susan Oliphant, Symposium Coordinator, Ontario Cancer Institute, 500 Sherbourne St., Toronto, Canada.

**First International Congress of the Italian Society of Immunopharmacology**
*10–11 October 1983* *Perugia University, Italy*
Dr. Carlo Riccardi, Scientific Secretary, Institute of Pharmacology, University of Perugia, Italy.

**Detection and Treatment of Minimal Residual Disease in Acute Leukaemia**
*12–14 October 1983* *Rotterdam, The Netherlands*
Dr. J.W. van der Velden, Dr. Daniel den Hoed Klinik/Rotterdam Radio Therapeutic Institute, P.O. Box 5201, 3008 AE Rotterdam, The Netherlands.

**UICC Workshop on Doctor Involvement in Public Education about Cancer**
*16–18 October 1983* *Kibbutz Shefayim, Israel*
David W. Reed, I.U.A.C., 3 rue de Conseil-Général, 1205 Geneva, Switzerland.

**Cancer Chemotherapy and Selective Drug Development**
10th International Symposium on the Biological Characterization of Human Tumours.
*24–28 October 1983* *Brighton, England*
CCHTI Secretariat, Institute of Cancer Research, Block E, Clifton Avenue, Belmont, Sutton, Surrey SM2 5PX, England.